# Research progress on the application of virtual simulation in medical education

**DOI:** 10.3389/fmed.2026.1864139

**Published:** 2026-07-17

**Authors:** Xiwang Jiang, Haichao Ge, Qianyong Wang

**Affiliations:** 1 Health Science Center, Ningbo University, Ningbo, China; 2 Department of Gastroenterology, The First Affiliated Hospital of Ningbo University, Ningbo, China

**Keywords:** basic medicine, clinical medicine, medical education, nursing medicine, virtual simulation

## Abstract

Medical education is fundamental to developing clinical competence, directly influencing care quality and patient safety. Traditional teaching models have long faced structural limitations, including scarce physical resources and restricted clinical exposure, which hinder the training of versatile professionals needed in modern health care. Virtual simulation (VS), by providing a high-fidelity, repeatable, and low-risk learning environment, offers a promising solution to these challenges. This narrative article reviews recent applications of VS in basic medicine, clinical medicine, and nursing education. Although existing evidence supports its educational benefits, most studies rely on short-term, small-sample designs and lack evaluation of long-term clinical transfer or cost-effectiveness. Persistent issues include high device heterogeneity and insufficient integration of nontechnical skills training. Future efforts should prioritize multicenter longitudinal research, platform standardization, and the incorporation of artificial intelligence to enable personalized adaptive learning—moving VS toward a core component of medical education.

## Graphical Abstract

**Figure:**
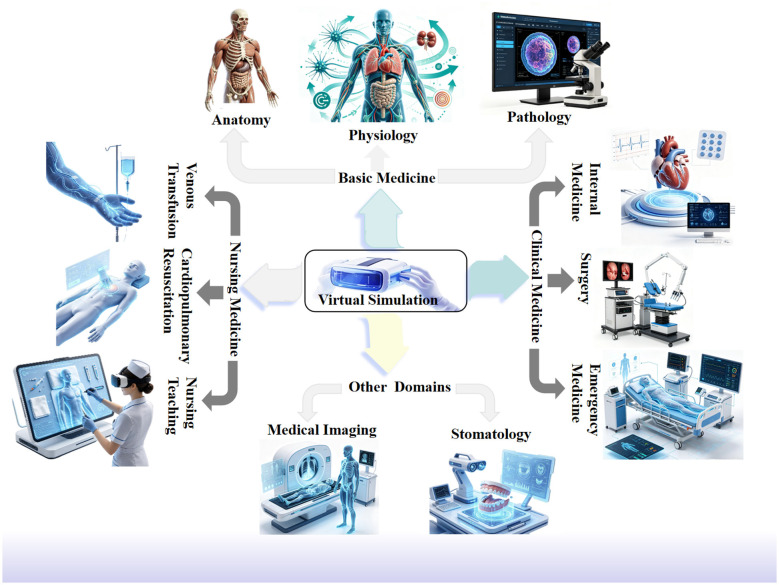

## 1 Introduction

Medical education operates as a complex system shaped by interactions among the learning environment, individuals, and institutional culture. A central goal is to prepare competent physicians for clinical practice (1, 2). More specifically, it aims to shape how learners think, perceive their profession, and act—a process often termed professional identity formation. Traditionally, this has involved not only transmitting core knowledge but also integrating social culture, professional norms, and organizational behavior standards (3). Through multiple pathways, medical education sustains and promotes health, prevents and treats disease, thereby contributing profoundly to human and societal wellbeing (4). Yet traditional medical education faces persistent practical challenges. In basic medicine, anatomy instruction relies heavily on cadaveric specimens, which are often in short supply, require specialized lab facilities, pose difficulties in long-term preservation, and raise ethical concerns (5, 6). In clinical surgery, the long-followed “see one, do one, teach one” model is constrained by limited exposure to diverse surgical cases, potential risks to patients during hands-on training, and insufficient structured practice time (7, 8). These shortcomings collectively weaken educational outcomes and restrict the quality of medical talent development.

Virtual simulation (VS) is an interactive, computer-based teaching strategy increasingly recognized in medical education. In practice, however, the boundaries between VS and related technologies—such as virtual reality (VR), augmented reality (AR), and virtual standardized patients (VSP)—remain blurred. These terms are often used interchangeably in the literature. The broader domain encompasses patient simulation, extended reality, mixed reality, and other emerging themes. The rapid advancement of modern computer simulation has given rise to numerous innovative teaching practices based on VS, thereby driving the diversification of medical education formats (9–13). In recent years, VS has been widely adopted across various case-based learning scenarios, including surgical skills training, emergency and pediatric emergency medicine, basic medical instruction, medical radiological imaging, puncture or catheter manipulation, and interdisciplinary medical education (9). By creating virtual anatomy labs and surgical simulation environments that do not rely on physical specimens or real patients, VS allows students to gain near-real operative experience while avoiding potential risks in practice. This effectively addresses the resource constraints of traditional medical education and is steadily emerging as a key direction for medical education reform (14, 15).

In recent years, researchers worldwide have extensively studied virtual simulation in medical education across basic medicine (16–18), clinical medicine (19–21), and nursing (22–24), generating substantial findings. Nevertheless, a systematic synthesis of this body of work remains lacking. This article therefore reviews recent research in the field (Table 1), clarifying the current state of application, its strengths and limitations, and potential directions for improvement, with the goal of informing clinical teaching practice.

**Table 1:** The application of virtual simulation in various medical education fields.

Field	Subfield	Research	Reference
Basic medical	Anatomy	Virtual dissection (Pirogov system) VS. traditional autopsy	(30)
		Augmented reality (AR) and virtual reality (VR)	(31)
		Virtual reality (VR)-based learning VS. traditional learning	(32)
		Virtual interactive 3-dimensional models (VI3DM) VS. 2-dimensional photographs	(33)
	Physiology	Virtual reality (VR) incorporation into the practical session	(34)
		High-fidelity mannequin (iStan) in a cardiovascular physiology course	(38)
	Pathology	Virtual Microscopy e-learning modules (VM-eLM)	(42)
		An E-learning platform (PathoDiscovery)	(43)
Clinical medical	Internal medicine	A longitudinal simulation curriculum	(48)
		Hybrid simulation training group VS. waitlist control group	(49)
	Surgery	A self-developed virtual simulation teaching platform VS. traditional classroom teaching and surgical video playback	(52)
		Virtual reality (VR) model+3-dimensional (3D) printed model VS. 3D printed model	(53)
	Emergency medicine	Virtual patient simulation (VPS) VS. in-person simulation (IPS)	(57)
		Extended reality (XR)/virtual reality (VR)-based training	(58)
Nursing medical	Venous transfusion teaching	Intra-venous injection virtual reality simulator (IIVRS) training	(22)
		Virtual reality technology VS. intravenous injection arm model	(63)
	Cardiopulmonary resuscitation	Virtual simulation+low-fidelity simulation VS. low-fidelity simulation	(23)
		Virtual reality (VR) group VS. traditional simulation group	(66)
	Clinical nursing teaching	Virtual reality (VR) learning group VS. traditional learning group	(69)
		A two-week mobile web-based training programme VS. a training booklet	(70)
Other domains	Stomatology	Simodont VS. artificial teeth (AT) mounted within phantom head (PH)	(69)
		A Virtual reality (VR) simulator+acrylic teeth VS. acrylic teeth	(73)
	Radiological imaging	A virtual reality (VR) group VS. a physical simulation group	(77)
		An interventional radiology (IR) simulator using a virtual reality system (the VR–IR simulator) VS. conventional verbal explanations and educator demonstrations	(78)

## 2 The application of virtual simulation in basic medical education

Basic medicine underpins medical knowledge and clinical practice, playing an irreplaceable role in medical education. The discipline focuses on human structure, organ function, and the mechanisms that maintain homeostasis, thereby providing the theoretical foundation for understanding disease pathogenesis. It also serves as a key source for medical students as they build their clinical knowledge system. Foundational courses such as anatomy, physiology, biochemistry, and pathology establish a grasp of normal structure and function, while pathophysiology and diagnostics offer the necessary basis for designing treatment pathways (25, 26).

### 2.1 The application of virtual simulation in anatomy

Anatomy is a core discipline in biomedical education, examining the typical morphology and structure of the human body from both macroscopic and microscopic levels. It serves as an essential foundation for undergraduate health sciences courses and a common pillar for understanding many subsequent clinical subjects (27, 28). Traditional teaching methods—such as student-led dissection, specimen observation, and model use—often fail to bridge basic knowledge and clinical practice, leading students to memorize mechanically without deep understanding. This restricts their clinical reasoning, spatial cognition, and ability to handle real-world challenges (28, 29). Moreover, reliance on physical specimens brings additional problems: resource shortages, demanding laboratory conditions, and difficulties in sample preservation, all of which further undermine teaching effectiveness (5, 6). VS offers a new solution to these challenges. Several studies have applied it to anatomy teaching. Duc NT et al. (30) compared virtual dissection (Pirogov system) with traditional autopsy for knowledge acquisition. Both groups showed significant improvement (virtual: 4.3 ± 1.65 to 5.2 ± 1.75, *p* < 0.001; traditional: 4.2 ± 1.92 to 5.1 ± 1.64, *p* < 0.001), with no between-group difference (*p* = 0.656). Gurses ME et al. (31) evaluated neuroanatomy training using based on VS. Test accuracy rose from 7.5/10 to 9.7/10 (*p* < 0.001) for residents and from 4.8/10 to 8.7/10 (*p* < 0.001) for students. User feedback unanimously indicated that VS greatly enhanced the learning experience. Additional work by Hammouda SB (32) and Chauhan P (33) further supports the potential value of VS in anatomy teaching.

In the VS studies of anatomy reviewed here, Duc NT et al. (30) found no difference in short-term knowledge gains between the Pirogov interactive platform and traditional autopsy, though students appreciated the visual advantages. Chauhan P (33) reported that a 3D interactive virtual model (VI3DM) significantly improved knowledge scores compared to 2D images. Both Gurses ME et al. (31) and Hammouda SB et al. (32) showed marked pre-to-post test improvements but lacked direct comparisons with conventional methods. None of these studies tracked long-term skill retention or clinical translation. Aside from Duc NT's et al. (30) reported equipment cost ($150,000–$170,000), no systematic cost-effectiveness analysis was provided. Scalability is limited by reliance on VS headsets, software, and network access. In terms of course integration, virtual tools can supplement cadaveric dissection, but implementation barriers such as motion sickness and equipment familiarity remain. These studies suggest that VS can address some challenges in traditional anatomy education—including limited specimen resources, weak clinical connections, and difficulty visualizing spatial relationships—while enhancing learners' knowledge mastery and experience, thereby offering a scalable reform pathway for basic medical education.

### 2.2 The application of virtual simulation in physiology

Physiology takes functional interpretation as its core approach. It seeks to clarify how each component of an organism contributes to the whole and to reveal the fundamental principles of life by integrating information across levels and systems. The discipline provides function-based explanations, describes normal and pathological states, maintains the organism's integrity, and uses unifying concepts such as homeostasis. Physiology not only defines the “items to be explained” in biomedicine but also integrates findings from fields like molecular biology into a coherent picture of organismal function. It bridges micro and macro levels in evolutionary biology, offering key support for understanding purpose, agency, and causality (34, 35). Traditional physiology teaching relies mainly on lectures. Although this mode conveys information efficiently, student engagement is low, and it struggles to overcome common conceptual barriers in the field. Learners often end up with fragmented knowledge, have difficulty transferring principles to new contexts, and show weak integrative reasoning. In a teacher-centered environment, students passively listen and take notes, with few opportunities for questioning or interaction. This leads to declining attention, reduced interest, weakened motivation, and conceptual errors that tend to persist over time (36, 37). In recent years, educators have begun introducing virtual simulation into physiology teaching. Lim JJ et al. (18) integrated VS into cardiopulmonary physiology lab courses for medical students and assessed the learning experience using questionnaires and interviews. 60% of respondents reported clear learning goals and received VS support. Clarity of learning objectives correlated positively with VS's educational effect (2022 cohort: unstandardized B = 0.683, *p* < 0.001, R^2^= 0.460; 2023 validation cohort: B = 0.661, *p* < 0.001, R^2^ = 0.682). Separately, Zheng J et al. (38) used a high-fidelity mannequin (iStan) in a cardiovascular physiology course. Compared with 2016 and 2017, the rate of complete mastery of cardiovascular physiology concepts rose significantly in 2018. Logistic regression showed that the odds ratio for full mastery in 2018 versus 2017 was 4.068 (a 306.8% increase, *p* < 0.001) and versus 2016 was 4.319 (*p* < 0.001). The average time students spent per question dropped to 79.49 s in 2018, down from 193.40 s in 2017 and 171.78 s in 2016 (*p* < 0.001). Student feedback indicated a positive learning experience (mean 4.41/5) and high perceived value (mean 4.20/5) (38).

In the VS studies of physiology reviewed here, Lim JJ et al. (18) used a mixed-methods approach, reporting that students' positive perceptions and clarity of learning goals correlated strongly with perceived VS utility. However, the study lacked objective knowledge tests and a control group, placing its evidence level relatively low. Zheng J et al. (38) employed historical controls, showing that the simulation group achieved a 306% higher concept mastery rate and reduced test time, but the design was not randomized, and long-term retention or clinical reasoning transfer was not assessed. Neither study reported cost-benefit or scalability analyses. In terms of course integration, VS served as a one-time supplement, whereas high-fidelity simulation was embedded across multiple teaching stages. Implementation barriers included device dependence, motion sickness, and student distraction. Together, these studies suggest that VS can effectively address shortcomings of traditional lecture-based physiology teaching—such as passive learning, difficulty integrating concepts, and weak transfer and application of skills—thereby providing strong support for cultivating deep functional reasoning and clinical integration abilities.

### 2.3 The application of virtual simulation in pathology

Pathology analyzes tissues, organs, body fluids, and autopsy findings to uncover disease mechanisms and inform clinical decisions and treatment strategies, making it a cornerstone of medical research and diagnosis (39). Traditional pathology teaching relies on glass slides and light microscopes, which pose problems such as limited learning resources and high time demands. Moreover, the lecture-dominated format overemphasizes memorization over clinical reasoning, and the use of real cases remains insufficient, hindering students' development of diagnostic thinking (40, 41). Virtual simulation offers a practical way forward. Cubillos P et al. (42) developed a virtual microscope—based online learning module for gynecologic cytopathology (VM-eLM). After training, average test scores rose by 16%, and the false-negative rate dropped by 88%. Among novices, false negatives decreased by 78% (*p* < 0.001); experienced users eliminated false negatives entirely. Recognition of low-grade and high-grade squamous intraepithelial lesions (LSIL, HSIL) improved by 31% and 50%, respectively (*p* < 0.001). Participants rated the module as novel, supportive of remote training, timely in feedback, and high in whole-slide image quality. Separately, Buma AIG et al. (43) designed and evaluated PathoDiscovery, an interactive online tool for teaching tumor-related pathophysiology and histopathology to medical students. Student ratings of the online module increased from 6.8 to 7.4 out of 10. In the improved academic year, 90% of students found the module structure logically clear, 57% considered the difficulty level appropriate, 76% agreed the modules matched the learning objectives, and 78% believed the modules supported knowledge development. The tool received positive feedback as a dynamic resource for blended learning (43).

In the VS studies of pathology reviewed here, Cubillos P et al. (42) used objective clinical indicators and found that false negatives dropped by 78% in the inexperienced group, while correct diagnosis of HSIL increased by 50%. In contrast, Buma AIG et al. (43) relied solely on students' subjective feedback and reported no objective knowledge or skill measures, resulting in a lower level of evidence. Neither study tracked long-term skill retention or transfer to real diagnostic scenarios. Cost-effectiveness was not systematically evaluated. Cubillos P et al. (42) faced technical barriers such as large WSI file sizes and long loading times, whereas Buma AIG et al. (43) was restricted by GDPR compliance and could not provide personalized feedback. In terms of course integration, both served as supplements to traditional teaching, but scalability is limited by network bandwidth, storage costs, and investments in platform development and maintenance. These studies suggest that VS can help overcome bottlenecks in traditional pathology education—such as limited physical slide resources and insufficient diagnostic reasoning training. This technology improves lesion recognition accuracy, reduces false-negative rates, and strengthens students' ability to integrate knowledge structures with clinical logic, offering an efficient, scalable pathway for cultivating pathological diagnostic thinking.

In summary, VS is gradually being integrated into medical basic courses. In anatomy, VS ease the shortage of specimen resources and the challenge of spatial cognition while improving knowledge acquisition. In physiology, VS and high-fidelity simulators shift the focus from passive lectures to active exploration, boosting learning efficiency. In pathology, virtual microscopes and interactive modules lower false-negative rates in lesion recognition and strengthen diagnostic reasoning. Overall, VS not only addresses the limitations of traditional teaching—insufficient resources and weak clinical connections—but also offers a high-fidelity reform pathway for basic medical education, one that promotes knowledge transfer through measurable learning gains. Future work should focus on assessing long-term educational outcomes and optimizing blended teaching models.

## 3 The application of virtual simulation in clinical medical education

Clinical medicine is more than an empirical science focused on diagnosing and treating disease. It also embodies practical wisdom—making value judgments under uncertainty, assuming ethical responsibilities, and building intersubjective caring relationships grounded in trust and understanding (44).

### 3.1 The application of virtual simulation in internal medicine

Internal medicine is one of the most valuable specialties in medicine, dedicated to preventing, diagnosing, and treating adult diseases across areas such as rheumatology, immunology, endocrinology, infectious diseases, and respiratory medicine. Its core task is to integrate history taking, physical examination, and ancillary tests to assess disease presentations and formulate treatment plans (45). Traditional internal medicine teaching emphasizes factual memorization, which struggles to keep pace with the exponential growth of medical knowledge. Learners often feel overwhelmed by large volumes of information, leading to superficial conceptual understanding and burnout. The teacher-centered, passive-acceptance model dampens learning motivation, gives insufficient attention to critical thinking and problem-solving, and the hierarchical structure further suppresses independent thinking and creativity (46, 47). Grebennikov S et al. (48) conducted a nine-month multimodal simulation program for internal medicine residents, covering central venous catheterization, thoracic and abdominal paracentesis, point-of-care ultrasound (POCUS), and clinical simulation cases. After the course, 98.6% of participants reported that the simulation provided new information or helped organize existing knowledge. All trainees felt more confident in performing procedures. 98.9% were more confident distinguishing normal from abnormal ultrasound findings, and 94.7% believed the clinical simulation improved their diagnostic ability. In a randomized controlled trial, Daly M et al. (49) used simulated patients wearing a novel auscultation vest for three counseling assessments on heart valve disease. Total scores rose from 39.6% on the first attempt to 63.6% on the third—a relative increase of 60.6% (*p* < 0.001). On the second assessment, the training group scored significantly higher than controls (54.2 ± 7.1% vs. 45.6 ± 9.2%, *p* < 0.001). Inter-assessor reliability was excellent (ICC 0.885–0.996), and students' self-assessed confidence and readiness also improved markedly.

In the internal medicine simulation studies reviewed here, Grebennikov S (48) used a single-group before-after design relying on self-reports and a few instructor evaluations, showing increased confidence but lacking objective skill endpoints and a control group. Daly M et al. (49) employed a randomized controlled, blinded design, demonstrating that mixed simulation significantly improved objective scores such as those for cardiac physical examination. Neither study tracked long-term skill retention or clinical behavior change, nor reported cost-effectiveness. Regarding scalability, vertical courses require ongoing resource investment, while hybrid simulation depends on wearable devices and SP training. In course integration, Grebennikov S et al. (48) replaced one-third of lectures with simulations, whereas Daly M et al. (49) used simulation as an add-on module. Implementation obstacles included time constraints, equipment complexity, and pandemic-related sample loss. These studies suggest that multimodal simulation technology—by providing a safe, repeatable clinical practice environment—enhances learners' operational confidence, diagnostic ability, and comprehensive assessment performance, thereby helping to bridge the gap between traditional teaching and clinical practice.

### 3.2 The application of virtual simulation in surgery

Surgery is a discipline in which technical skill directly affects patient prognosis, influencing short-term complications and mortality. The core goal is to ensure patient safety and improve outcomes through objective assessment of surgical performance (50). Traditional surgical teaching follows the “see one, do one, teach one” apprenticeship model. This approach does not emphasize standardized basic techniques and lacks real-time intraoperative feedback. Resident autonomy and exposure to diverse cases are limited. Training tends to prioritize quantity over quality. Restricted duty hours further reduce operating room time and total case volume, thereby diminishing opportunities for direct mentoring and trust-building (8, 51). Liu J et al. (52) compared VS teaching (experimental group) with traditional teaching (control) using knowledge tests, virtual operations, and questionnaires. The experimental group scored significantly higher on anatomical knowledge (79.44 ± 7.43 vs. 73.95 ± 6.25, *p* < 0.001) and virtual appendectomy (74.06 ± 8.34 vs. 71.18 ± 6.52, *p* = 0.031). Learning satisfaction was also higher in the experimental group (83.08 ± 9.74 vs. 76.03 ± 5.36, *p* < 0.001) (52). Hoglund BK et al. (53) compared two groups: one received VS training before using 3D-printed models for surgery, the other used only 3D-printed models. The VS-trained group showed significantly better outcomes across several measures: proportion of craniotomy size not requiring adjustment (70% vs. 20%, 95% CI 23.3%-76.7%, *p* < 0.01), correct osteotomy sequence rate (90% vs. 30%, *p* < 0.01), surgical completion rate (85% vs. 40%, 95% CI 18.4%-71.6%, *p* < 0.01), and use of skull landmarks (95% vs. 55%, 95% CI 16.2%-63.8%, *p* < 0.01). 95% of participants rated the model as having sufficient anatomical fidelity.

In the surgical VS studies reviewed here, Liu J (52) used an RCT design and found that VS improved anatomical knowledge and virtual surgical operations but did not significantly enhance fine motor skills such as suturing and knotting. Hoglund BK (53) employed objective performance indicators, showing that VS pre-training significantly improved the accuracy, sequence, and time efficiency of craniotomy, with 3D-printed models costing $47.52. Neither study tracked long-term skill retention or clinical translation. Regarding cost-effectiveness, Hoglund BK (53) explicitly reported low costs, while Liu J et al. (52) provided no such data. Scalability is limited by lack of haptic feedback and reliance on 3D printing or VS devices. In course integration, VS serves as a supplement to traditional teaching. Implementation barriers include beginners' adaptation to spatial manipulation and the absence of real tissue sensation. These studies suggest that VS can address shortcomings of the traditional surgical apprenticeship model—such as insufficient standardization, lack of real-time feedback, and limited surgical exposure—while improving learners' anatomical knowledge, technical skills, procedure completion rates, and learning satisfaction, thereby offering a new pathway for enhancing patient safety and optimizing surgical training quality.

### 3.3 The application of virtual simulation in emergency medicine

Emergency medicine provides timely and accurate care for any patient, in any setting, for any problem. It has evolved into a systematic care system with a distinct clinical practice that spans from pre-hospital to community settings. Its functions include managing time-sensitive conditions, performing resuscitation, monitoring symptoms, and collaborating with public health to continuously improve the quality of public care (54). Traditional emergency medicine teaching faces several limitations: limited hands-on opportunities, a disconnect between theory and practice, lack of environmental and psychological realism in simulation training, and reliance on subjective feedback with insufficient attention to long-term knowledge retention (55, 56). Low MJW et al. (57) compared junior emergency physicians' performance in sepsis and trauma resuscitation training using VS versus on-site simulation. No significant differences emerged between the two groups for sepsis (overall score difference 2.0, 95% CI−1.4 to 5.4, Cohen's d = 0.38) or trauma (overall score difference-0.9, 95% CI−4.1 to 2.3, Cohen's d = 0.19), including cognitive and non-cognitive subscores. Separately, Einloft J et al. (58) used a VS application with wireless head-mounted displays to assess the acceptability and learning outcomes of extended reality (XR) in emergency teaching. Among students, 84% found the device intuitive and easy to use, 83% reported high immersion, 91% considered VS an effective learning tool, and 94% wished for more such interactive teaching.

In the emergency medicine VS studies reviewed here, Low MJW et al. (57) used an RCT design and found no significant differences between VS and in-person simulation for sepsis and trauma resuscitation skills (Cohen's d = 0.19–0.38), though learner satisfaction was significantly lower in the VS group. Einloft J et al. (58) conducted a single-arm pre-post study relying solely on subjective self-reports; despite high reported acceptance, the study lacked objective skill measures and a control group, resulting in relatively low evidence. Neither study tracked long-term skill retention or clinical behavior change. Regarding cost-effectiveness, Einloft J et al. (58) compared two types of head-mounted displays but did not calculate total investment, while Low MJW et al. (57) did not report costs. Scalability is limited by device dependency, motion sickness, and network conditions. In terms of course integration, Low MJW et al. (57) used VS as a single add-on module, whereas Einloft J et al. (58) embedded VS into a required course with student mentoring, achieving deeper integration. Implementation barriers included insufficient active engagement in the VS group, and about one-third of students in the VS group felt the simulation lacked realism. Future research should prioritize RCTs that include objective endpoints and long-term follow-up. These studies suggests that VS effectively addresses shortcomings of traditional emergency medicine teaching—such as limited hands-on opportunities and insufficient environmental and psychological authenticity—offering a new pathway for training in time-sensitive diseases and resuscitation procedures.

In summary, VS has been gradually integrated into teaching across clinical specialties. In internal medicine, randomized controlled trials confirm that multimodal simulation improves trainees' comprehensive assessment performance and diagnostic confidence. In surgery, combining VS with 3D printing overcomes limitations of the traditional apprenticeship model, enhancing objective measures such as surgical completion rates, osteotomy sequence accuracy, and learning satisfaction. In emergency medicine, virtual patient simulation achieves outcomes comparable to on-site simulation, and learners consistently recognize its immersion and educational value. By providing a safe, repeatable, and standardized clinical practice environment, VS effectively addresses shortcomings of traditional teaching in hands-on opportunities, real-time feedback, and clinical reasoning. It offers an evidence-based pathway for developing clinicians who possess both technical skill and ethical judgment. Future efforts should further integrate artificial intelligence and mixed reality to promote the construction of a full-cycle skills assessment system.

## 4 The application of virtual simulation in nursing medical education

Nursing medicine is a distinct knowledge system that integrates theory, research, and practice, with human wellbeing at its core. It encompasses health promotion, disease prevention, individual care, rehabilitation, and influence on public health policy. Guided by its own epistemological framework and knowledge models, nursing emphasizes compassionate care, empathy, and active listening. It continuously generates new knowledge from observation, feeling, and real experience. Nursing medicine is neither a closed nor static pure science; rather, it blends scientific knowledge with humanism, responds dynamically to social issues, and provides coordinated care for individuals and groups (59, 60).

### 4.1 The application of virtual simulation in venous transfusion teaching

Venous transfusion is one of the most fundamental and core procedures in clinical nursing. Traditional teaching relies largely on plastic prosthetic arm models, which come with several problems: an artificial environment, fixed rather than variable conditions, vessel positions that differ from real patients, lack of comprehensive experience with actual patients, and high cost of models as consumables (61, 62). Chang YY et al. (22) used the intravenous injection virtual reality simulator (IIVRS) with undergraduate nursing students in a pre-test/post-test design. Intravenous knowledge scores rose significantly from 3.08 to 4.96 (*p* < 0.001). Students reported high acceptance of VS (mean 4.65/5) and high learning motivation (mean 4.69/5), indicating that VS can serve as an effective supplementary tool for nursing skills teaching. Yildiz H et al. compared a 3D VS platform for venous catheterization with a traditional intravenous injection arm model. The experimental group scored significantly higher on the skill checklist (88.94 ± 9.22 vs. 65.13 ± 11.12, *p* = 0.001), suggesting that VS positively affects nursing students' mastery of intravenous catheterization and infusion skills (63).

Both studies confirmed that VS improves short-term intravenous infusion skills in nursing. However, Yildiz H et al. (63) used an RCT design, providing stronger evidence than Chang YY's et al. (22) single-group pre- and post-test design. Neither study tracked long-term skill transfer, reported cost-effectiveness, or examined cross-institutional scalability. Implementation barriers centered on lack of haptic feedback and insufficient operational smoothness, suggesting that VS should supplement rather than replace traditional training. The available evidence indicates that VS can address partial limitations of conventional nursing education—such as the unrealistic feel of plastic arm models, vascular positions that differ from clinical settings, and high costs. Both empirical studies consistently show that VS-based intravenous infusion training improves nursing trainees' knowledge, technical skills, and learning motivation, thereby offering an efficient method for teaching essential nursing procedures.

### 4.2 The application of virtual simulation in cardiopulmonary resuscitation

Cardiopulmonary resuscitation (CPR) is a core emergency skill in nursing that directly affects patient survival. Traditional CPR teaching faces several issues: rapid skill decay, high demands on personnel and time that are difficult to sustain, and a lack of immersive experience and real-stress environment. These shortcomings lead to poor quality of chest compressions after training and limit overall teaching effectiveness (64, 65). Li Y et al. (23) compared two CPR teaching approaches: an experimental group using blended learning (online VS plus offline low-fidelity simulation) and a control group using only offline low-fidelity simulation. The experimental group scored significantly higher on CPR skills (91.56 ± 3.426 vs. 84.28 ± 3.221, *p* < 0.001) and on total self-directed learning ability (106.97 ± 9.107 vs. 98.19 ± 12.235, *p* = 0.001). The experimental group also outperformed controls on three critical thinking subscales—truth-seeking, analytical thinking, and confidence (all *p* < 0.05). Bodur G et al. (66) compared VS-based headsets with traditional mannequins in CPR training. The VS group performed significantly better on several Objective Structured Clinical Examination (OSCE) steps, including chin lift, airway assessment, and breathing judgment (*p* < 0.05). VS also supported the abstract conceptualization learning style (*p* = 0.054). These findings suggest that VS enhances both motor skills and cognitive engagement in CPR, with even better results when combined with traditional methods.

A critical comparison of two VS studies on CPR training in nursing reveals important differences. Li Y et al. (23) used an RCT design comparing blended learning with VS to traditional instruction alone, finding that the experimental group performed significantly better in CPR skills and self-directed learning. Bodur G (66) directly compared VS with traditional simulation, reporting no difference in knowledge gains between groups; however, VS led to superior performance on several key psychomotor skills, whereas traditional simulation promoted greater autonomous learning ability. Neither study tracked long-term skill retention or clinical translation. Cost-effectiveness was not reported, but VS requires headsets and software licenses, and scalability is limited by device availability and physical space. In terms of course integration, VS is better suited as a supplement rather than a replacement. Implementation obstacles include dizziness, eye fatigue, and VS's limited impact on abstract conceptualization. Together, these studies suggest that VS can address partial challenges in traditional CPR teaching—such as rapid skill decay, high resource demands, and the lack of a realistic stress environment. VS helps improve CPR skill scores, self-directed learning abilities, and standardization of key procedural steps, offering a new approach to optimizing emergency nursing education.

### 4.3 The application of virtual simulation in clinical nursing teaching

Nursing clinical teaching represents the core link where nursing education integrates deeply with clinical practice. Traditional approaches—including teaching ward rounds—rely largely on preview-based instruction and bedside practice. Yet clinical settings often fail to provide consistent opportunities for practicing high-difficulty or low-frequency events. Limited patient volume and space further restrict student participation, which in turn greatly hinders timely, structured practice of core nursing skills (67, 68). Hung HY et al. (69) evaluated a VS teaching model for immediate neonatal care (INC). They compared a VS learning group with a traditional learning group on knowledge, skill confidence, procedural accuracy, and completion time. No statistically significant differences emerged between the two groups in knowledge, confidence, or accuracy. However, the VS group's average practice time (10.3 min) was substantially shorter than that of the traditional group (35 min), suggesting that VS can serve as a supplementary tool to traditional teaching by offering a convenient, repeatable practice platform. Oh JW et al. (70) used a VS-based smartphone application to teach patient safety management to nursing students. The experimental group used the VS and the control group received a training manual. Outcomes included knowledge, attitude, and confidence. The experimental group showed significantly greater gains in patient safety management knowledge (from 11.68 to 18.55 points, *p* < 0.001), attitude (from 3.38 to 4.01 points, *p* < 0.005), and confidence (from 3.26 to 3.53 points, *p* < 0.030).

A critical comparison of two VS studies in clinical nursing teaching reveals distinct approaches and findings. Hung HY (69) used a non-inferiority design for VS training in immediate neonatal care, finding no significant differences in knowledge, skills, or confidence between VS and traditional instruction, though VS significantly reduced learning time. In contrast, Oh JW (70) reported that VS applied to patient safety management led to significantly better outcomes in knowledge, attitude, and confidence than a traditional paper-based method. Neither study tracked long-term skill transfer or clinical behavior change, and neither provided a cost-effectiveness analysis. Scalability is limited by small sample sizes and device dependency. In terms of course integration, VS is mostly used as a supplementary practice tool, but technical obstacles such as motion sickness and operational smoothness remain to be addressed. Overall, these studies suggest that VS can effectively address shortcomings of traditional nursing clinical education—including insufficient practice opportunities for high-difficulty or low-frequency events, and constraints imposed by patient availability and physical space. VS can also shorten skill practice time and enhance learners' knowledge, attitude, and confidence in safety management, offering a convenient, repeatable, and efficient pathway for clinical teaching.

VS has been gradually embedded across multiple areas of nursing education. In intravenous infusion training, VS simulators improve learners' knowledge, technical skills, and motivation. In CPR teaching, combining online VS with hands-on practice or using head-mounted VS devices leads to better skill scores, self-directed learning, and standardization of key steps compared to traditional methods. In clinical nursing education, VS platforms reduce skill practice time while enhancing knowledge, attitudes, and confidence in patient safety management. Additionally, VS addresses persistent challenges in traditional nursing education—such as model inaccuracy, resource limitations, and lack of realistic stress—by offering a repeatable, high-fidelity, low-risk immersive training environment. This technology provides an efficient, evidence-based, and scalable pathway for preparing nursing professionals who combine strong clinical skills with humanistic care.

## 5 The application of virtual simulation technology in education of other domains

Beyond its established applications in basic medicine, clinical medicine, and nursing medicine, virtual simulation has also shown meaningful value in specialized fields such as stomatology and radiological imaging. Although current research remains relatively limited, the potential for further development is considerable.

### 5.1 The application of virtual simulation in stomatology

Stomatology is a clinical specialty focused on diagnosing and treating oral and maxillofacial diseases, encompassing patient oral health care and the management of related disorders and functional impairments (71). Traditional teaching has several limitations, for example, oral anatomy instruction, relies heavily on lectures with one-way communication, limited interaction, and low student engagement. The course also lacks integration with other clinical content. Enlarged carving materials may distort students' understanding of true anatomical proportions (72). Slaczka DM et al. (73) compared a tactile VS simulator (Simodont) with traditional artificial teeth for pulpotomy training on mandibular first molars. The improvement rate was 23% in the VS group and 39% in the control group, with no statistically significant difference (*p* = 0.315). Questionnaire responses indicated that Simodont helped students visualize pulpal anatomy in three dimensions and was well accepted. The training effect was comparable to that of traditional artificial teeth, supporting its use as an adjunct for pulp opening training. Heidaridarani MM et al. (74) evaluated the DentaSim VS simulator versus a control condition for preparing Class I cavities on mandibular first molars. The VS group showed significantly greater overall performance improvement than controls (*p* < 0.05). In the first training stage, the VS group made significant progress on 5 of 9 scoring criteria; in the second stage, it outperformed controls on 6 criteria. The incidence of serious errors dropped sharply in the VS group (*p* = 0.000), whereas the decrease was smaller in the control group (*p* = 0.006). In the second stage, serious errors continued to decline significantly in the VS group (*p* = 0.004) but showed no change in controls (*p* = 1.000). These findings suggest that VS simulation can improve the quality of pre-clinical restorative dentistry training and serve as a useful adjunct in dental education.

A critical comparison of two VS studies in dental education reveals divergent findings. Slaczka DM et al. (73) found no significant difference in skill improvement between the Simodont tactile VS system and traditional training. In contrast, Heidaridarani MM (74) reported that the DentaSim VS plus traditional group outperformed traditional training alone on multiple measures, with the traditional group reaching a learning plateau after 4 h. These discrepancies may stem from differences in task complexity, VS exposure time, and feedback design. Neither study tracked long-term skill retention or clinical translation, and neither reported cost-effectiveness. Scalability is limited by high equipment costs (e.g., Simodont at roughly tens of thousands of dollars), ongoing concerns over tactile fidelity, and motion sickness. These studies suggest that VS can address key limitations of traditional dental teaching—such as one-way instruction, limited interaction, and distorted understanding of anatomical proportions—offering an efficient, repeatable tool for supporting dental psychomotor skills training.

### 5.2 The application of virtual simulation in radiological imaging

Radiological imaging is a discipline that performs qualitative analysis using CT, MRI, and PET-CT to assist diagnosis, predict prognosis, and guide treatment decisions (75). Traditional teaching relies mainly on lecture-based instruction. While this mode conveys information efficiently, it is limited by passive learning, lack of real-time interaction, and difficulty maintaining student engagement. Teaching effectiveness is also influenced by case availability and variability across different radiologist instructors (76). Rowe D and colleagues compared a VS simulation group with a traditional physical simulation group, evaluating learning outcomes via OSCE after training on various X-ray projection positions. The VS group performed significantly better on several metrics: OSCE duration (912.2 vs. 1062.1 s), number of device movements (29.8 vs. 52.5), number of incorrect movements (3.6 vs. 14.6), and number of incorrect postures (all *p* < 0.01). These findings suggest that VS simulation holds potential value in radiological imaging education (77). Mitani H et al. (78) assigned participants to a conventional teaching group (traditional explanation and demonstration) or a virtual simulation interventional radiology (VS-IR) simulator group. After training, both groups completed a transarterial chemoembolization (TACE) test for hepatocellular carcinoma using an VS-IR simulator. The VS-IR simulator group used significantly less contrast agent (28.0 ml vs. 40.0 ml, *p* < 0.01) and achieved significantly higher technical operation scores (36 vs. 31 points, *p* < 0.01). The results indicate that the VS-IR simulator can reduce contrast agent use during interventional procedures and improve technical skill.

A critical comparison of two VS studies in radiological imaging reveals distinct performance outcomes. Rowe D et al. (77) found that the VS group significantly outperformed the traditional simulation group on OSCE measures of operation time, equipment movement errors, and patient positioning errors, though no difference was observed in exposure parameter settings. Mitani H et al. (78) reported that the VS group used less contrast agent and achieved higher technical scores, with no significant differences in operation time, fluoroscopy time, or skin dose. Neither study tracked long-term skill retention or clinical translation, and neither provided a cost-effectiveness analysis. Scalability is limited by hardware costs, VS-induced motion sickness, and equipment maintenance. Additionally, VS implementation must address issues of tactile loss and system stability. Overall, these studies suggest that VS can help overcome shortcomings of traditional radiological imaging education—such as passive learning, insufficient interaction, and limited case accessibility. VS simulation reduces operation time, equipment movements, and positioning errors, lowers contrast agent usage, and improves technical scores for interventional procedures, thereby offering an efficient, standardized pathway for imaging skills training.

## 6 Prospect

Medical education is central to training high-quality health professionals, directly affecting patient safety and public health standards. Traditional medical education has long faced structural shortcomings, such as shortages of physical specimens and limited clinical practice opportunities. These limitations make it difficult to meet modern medicine's demand for versatile, practice-ready clinicians (5–8). Virtual simulation, by creating high-fidelity, repeatable, low-risk learning environments, is gradually overcoming these barriers and offering a feasible pathway to reshape medical education. To date, VS is gradually solving the above-mentioned problems, it enhances knowledge acquisition, procedural skills, and clinical judgment, while also boosting learning motivation and self-directed learning. These features point to VS's potential as an effective supplement to—or even a partial replacement for—traditional medical education. Further support comes from Elendu C et al. (79), whose work reinforces the central role of simulation-based training (SBT) in medical education. Their findings show that SBT not only improves learners' technical skills but also offers clear advantages in developing non-technical competencies—including communication, teamwork, and clinical decision-making. Notably, the authors highlight the importance of a “safe learning environment” and “structured debriefing” for fostering deep learning, a finding that aligns closely with current educational theories of experiential learning and reflective practice. Although VS has shown clear teaching benefits in medical education, most current evidence comes from short-term, small-sample, single-center studies. Long-term tracking of skill retention and behavioral change in real clinical settings is lacking, and cost-benefit analyses remain nearly absent. Persistent implementation barriers include high costs and resource demands, differences in accessibility, inadequate teacher training, and challenges in technical maintenance and interoperability. Furthermore, VS has yet to systematically integrate non-technical competencies such as clinical communication, teamwork, and ethical decision-making. Future research should prioritize multicenter randomized controlled designs that include objective clinical endpoints and long-term follow-up to validate the added value of VS over traditional instruction. Second, open and interoperable platform standards are urgently needed to lower barriers between devices and software. Adaptive learning systems driven by artificial intelligence should also be developed, capable of dynamically adjusting task difficulty and feedback based on learners' real-time performance to enable personalized skill development.

Additionally, several countermeasures can help address the implementation obstacles outlined above. On cost and resources, institutions may adopt tiered equipment configurations aligned with teaching objectives, prioritize open-source or shared resources, and perform cost-effectiveness analyses. To bridge accessibility gaps, offline versions should be developed alongside inclusive designs for students with disabilities. For teacher training, programs should offer stratified instructional design and debriefing skill development, complemented by ready-to-use lesson plans to lower the adoption barrier. Regarding technical maintenance and interoperability, unified standards are needed to reduce the burden of device compatibility and upkeep. Overall, for VS to transition from innovative pilot projects to routine teaching, coordinated progress in resource allocation, faculty development, technical standards, and evidence-based research is essential to achieve effective implementation and tangible educational outcomes.

In summary, VS is shifting from an “auxiliary tool” to a “useful teaching platform”. Its systematic integration and advancement within medical education will provide key support for building a high-quality, highly accessible future learning system. Only through continued technological iteration—combined with collaborative innovation in educational concepts and assessment frameworks—can VS fully realize its transformative potential in medical talent development.
